# Sclerodermoid cutaneous metastases secondary to prostate adenocarcinoma

**DOI:** 10.1016/j.jdcr.2026.02.047

**Published:** 2026-03-06

**Authors:** Katherine De Jong, Eleanor Ostroff, JoJo Holm, Mara O’Connor, Caitlin Kearney, Madeline J. Hooper, Conor Driscoll, Victor L. Quan, Pedram Gerami, Xiaolong A. Zhou

**Affiliations:** a Department of Dermatology, Northwestern University Feinberg School of Medicine, Chicago, Illinois; b NYU Grossman School of Medicine, New York, New York; c Department of Urology, Northwestern University Feinberg School of Medicine, Chicago, Illinois

**Keywords:** cutaneous metastases, prostate adenocarcinoma, sclerodermoid, skin of color, skin oncology

## Introduction

Prostate adenocarcinoma (PCa) is the most common solid malignancy and the second leading cause of cancer-related death among American men.^1^ While cutaneous metastases of carcinomas have an overall incidence of 5.4%, PCa is among the rarest, with an incidence of 0.7%.^2^ The most frequently involved cutaneous sites for PCa include the lower abdomen, thighs, and scrotum, although lesions on the chest, back, and face have also been described.^1^ Morphology can be heterogeneous, with reports describing papules, nodules, zosteriform eruptions, polypoid growths, and hemorrhagic vesicles.^1^ These metastases generally occur late in the disease course and are associated with extremely poor prognosis (typically <1-year survival). Common treatments for cutaneous metastases of carcinomas include systemic and skin-directed therapies such as chemotherapy, radiotherapy, and intralesional therapy.^3^ However, standardized treatment guidelines for cutaneous metastases are lacking, making management challenging.

## Case report

A 74-year-old male with metastatic castrate-resistant PCa with osseous metastases on leuprolide presented with a 2-week history of acutely worsening right scapular and rib pain as well as new skin nodules.

He was initially diagnosed 3 years prior during an emergency room visit for lower back pain and 1 year of unintentional weight loss. Cross-sectional imaging demonstrated multiple sclerotic lesions in the bony pelvis, sacrum, and right femur with malignant spinal cord compression. Prostate-specific antigen (PSA) was >1420. Iliac bone biopsy confirmed metastatic PCa. His initial treatment regimen was comprised of androgen deprivation therapy with degarelix, darolutamide, and external beam radiation. Later, pembrolizumab, palliative radiation, and Lutetium-177 Prostate-Specific Membrane Antigen therapy were initiated to control disease progression. Four months prior to presentation, his PSA had declined to 2.93, and positron emission tomography/computed tomography showed a partial response with significantly decreased radiotracer uptake in the prostate and osseous metastases. There were no skin lesions at that time.

**Fig 1:**
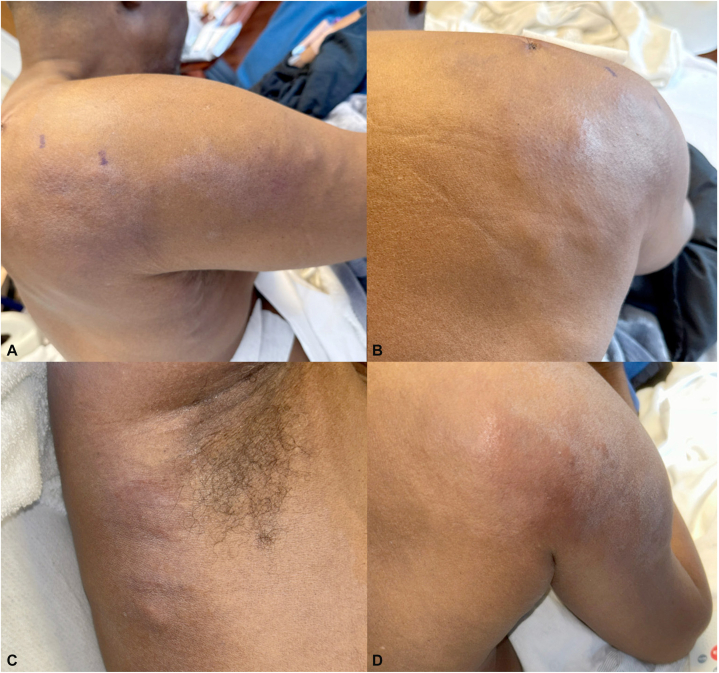
Clinical features. Multiple firm, thick, tender subcutaneous nodular and sclerotic plaques were noted on the right shoulder **(A**, **B** and **D),** upper arm **(A** and **D),** and axilla **(C)**. An ill-defined indurated pink plaque was also appreciated on the right posterior shoulder **(B** and **D)** and upper arm.

On presentation, his PSA had risen to 112. Physical examination demonstrated a Fitzpatrick skin type V individual with multiple firm, thick, and tender subcutaneous sclerotic and nodular plaques on the right shoulder, upper arm, and axilla (Fig 1, *A*-*D*). An indurated pink plaque measuring 25 × 16.5 cm was noted on the scapula and upper humerus (Fig 1, *A*, *B* and *D*). A 2.5-cm subcutaneous nodule was also noted on the midline spine.

**Fig 2:**
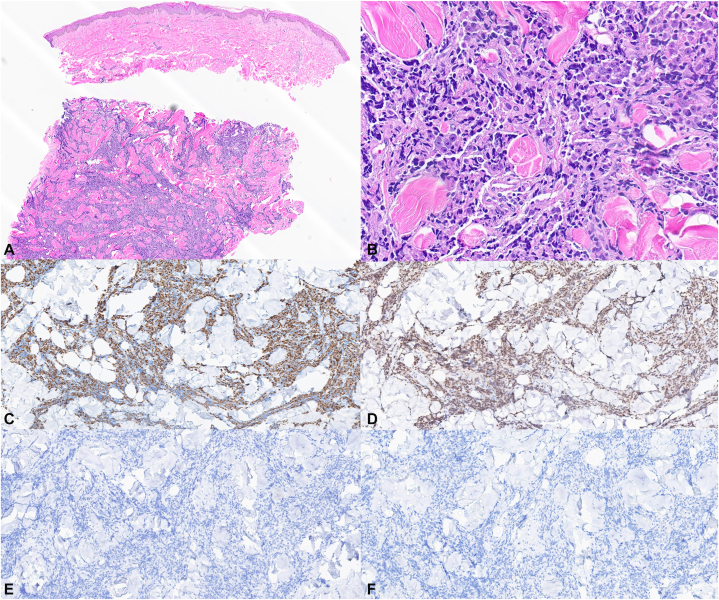
Histologic features. **A,** Punch biopsy of right shoulder plaque revealed dense sheets of atypical pleomorphic, hyperchromatic epithelial cells dissecting through thickened bands of collagen throughout the dermis (2×, H&E). **B,** Higher power demonstrated prominent nucleoli in the pleomorphic atypical cells. Immunohistochemistry revealed positive (20×, H&E) **(C)** AE1/AE3 (10×) and **(D)** NKX3.1 (10×) and negative **(E)** CK7 (10×) and **(F)** CK20 (10×).

Biopsy of plaques from the middle back and right shoulder revealed dense cords and sheets of atypical pleomorphic, hyperchromatic epithelial cells with prominent nucleoli dissecting through thickened bands of collagen throughout the reticular dermis (Fig 2, *A* and *B*). Tumor necrosis was present. AE1/AE3 and NKX3.1 were positive (Fig 2, *C* and *D*), while AMACR, p63, p40, CK7 (Fig 2, *E*), CK20 (Fig 2, *F*), SOX10, MART-1, S100, and PRAME were negative. Positron emission tomography/computed tomography revealed avid radiotracer activity in the right scapula, right axillary and supraclavicular lymph nodes, right pleura, and surrounding soft tissues.

The patient was diagnosed with sclerodermoid metastatic PCa and given palliative radiation (2 × 25 Gys) to his right shoulder lesions. Although his PSA climbed to 318 postradiation, he reported substantially decreased pain. The patient later succumbed to his disease 9 months after presentation.

## Discussion

This case represents a unique presentation of sclerodermoid metastases of PCa, with cutaneous lesions notably located near a site of prior osseous involvement. Sclerodermoid metastases can originate from internal adenocarcinomas (most commonly breast or larynx) due to the strong desmoplastic stromal response they induce in skin and subcutaneous tissue.^4^ The resulting fibroblast proliferation and collagen deposition leads clinically to sclerotic, thickened plaques and firm nodules. For PCa, the most common presentation of cutaneous metastases is of multiple nodules involving the suprapubic and thigh area. One prior case reported cutaneous PCa metastases that manifested as a sclerodermoid plaque on the chest.^5^ Our case demonstrated an unusual clinical manifestation of thickened, sclerotic plaques on the shoulder, axilla, and upper arm in a patient with skin of color.

Histological examination further supports a diagnosis of sclerodermoid metastases, as extensive dermal fibrosis with nests and cords of malignant cells were appreciated within the dermis. Immunohistochemistry also clearly identified cells of prostatic origin in our biopsies. NKX3.1 is highly specific to cells of prostate origin, while AE1/AE3 is specific for cells of epithelial origin and is commonly present in carcinomas.^6^ Negative CK7 and CK20 further reinforce the notion that the tumor cells did not derive from breast, lung, or gastrointestinal tissue.^6^

Precise survival rates for sclerodermoid metastases are not well established in medical literature due to their rarity. They are typically observed late in the disease course, serving as a poor prognostic indicator and thus difficult to treat. Literature on treatment of sclerodermoid metastases is almost entirely in association with cutaneous metastases of breast cancer, where they often can present as *carcinoma en cuirasse*. Most commonly, systemic therapies, mastectomies, or radiation (or a combination of the 3) are attempted, and the primary goal of treatment is palliation.^7^ Androgen deprivation therapy is the first-line systemic therapy for metastatic PCa.^8^ In castration-resistant PCa, additional systemic therapies including androgen receptor pathway inhibitors, taxane chemotherapy, and radioligand therapy (Lutetium-177 Prostate-Specific Membrane Antigen) have demonstrated improvement in survival and quality of life.^8^ There are currently no prospective studies that specifically address therapy for cutaneous metastases of any carcinoma.

Topical treatments for sclerodermoid metastases are uncommon given that most topical agents are unable to penetrate the fibrotic dermis and are inadequate for metastatic disease. However, limited case reports do suggest the utility of topical imiquimod, a toll-like receptor 7 agonist, for cutaneous metastases.^9^^,^^10^ While the effectiveness of radiotherapy for cutaneous metastases is debated, recent studies have found the combination of radiotherapy with other treatments, such as hormonal therapy or chemotherapy, may improve patient responses in *carcinoma en cuirasse*.^7^ Overall, treatment should be tailored to the individual’s disease burden, with a primary focus on symptom control. While imiquimod was considered for treating this patient, targeted radiation with androgen deprivation therapy was ultimately used with reduction in size of the sclerotic skin lesions and palliative success.

## Conflicts of interest

None disclosed.
